# An obvious antinomy, superior sagittal sinus thrombosis in a patient with immune thrombocytopenia: Case report and a review of literatures

**DOI:** 10.1097/MD.0000000000033412

**Published:** 2023-03-31

**Authors:** Yuhui Wang, Ge Zhang, Jinggang Ding

**Affiliations:** a Department of Critical Care Medicine, Sir Run Run Shaw Hospital Zhejiang University School of Medicine, Hangzhou, Zhejiang, China.

**Keywords:** case report, cerebral venous thrombosis (CVT), hemorrhagic disease, immune thrombocytopenia (ITP), thrombus

## Abstract

Immune thrombocytopenia (ITP) is an autoimmune disease with an increased risk of bleeding. However, in recent years, it has been reported that patients with this hemorrhagic disease have the risk of thrombosis and embolism.

**Patient concerns and diagnosis:**

The patient, in this case, was a young female who was diagnosed with ITP. When the platelet count was low, she had skin, mucosa, internal organs, and intracranial hemorrhage. In the process of ITP and hemostatic treatment, superior sagittal sinus thrombosis occurred when she was still bleeding.

**Interventions:**

She was given treatments for reducing intracranial pressure and controlling epilepsy.

**Outcomes:**

And then the embolectomy operation failed. It was suggested in this case that ITP patients with severe thrombocytopenia and bleeding tendency also have a risk of having thrombotic disease. We reviewed literatures regarding the mechanism of the simultaneous occurrence of 2 antinomy diseases and cerebral venous thrombosis.

**Lessons:**

There are many factors for ITP patients to have thrombosis involving ITP itself, its treatment and the patients’ constitution, medical history, and former medication. ITP is not only a hemorrhagic disease but also a thrombotic disease. Clinicians should be alert to the risk of thrombotic diseases in ITP treatment. Therefore thrombus monitoring and screening should be carried out, and early prevention or appropriate anticoagulant treatment should be selected, especially for patients with high risk.

## 1. Introduction

Immune thrombocytopenia (ITP) is an autoimmune disease characterized by antibody and cell-mediated destruction of platelets, accompanied by impaired platelet production and increased risk of bleeding.^[1]^ The patient in this case was diagnosed with ITP. During treatments, the platelet count increased slightly and decreased repeatedly. The patient had ecchymosis on both lower limbs and gingival bleeding, and the symptoms were constantly aggravating. Then vaginal and gastrointestinal bleeding appeared. During treatments, the patient had persistent headaches and vomiting. The brain CT suggested subarachnoid hemorrhage. The patient felt progressive aggravation of symptoms, and then she had epilepsy, unclear consciousness, and bilateral mydriasis, but the scope of bleeding on brain CT was not significantly expanded. Brain computed tomographic angiography (CTA) showed there was no obvious dilatation, stenosis, or tumor-like lesions in the cerebral arteries and their main branches. The display of superior sagittal sinus was poor, and there may be thrombosis. So brain computed tomographic venography (CTV) examination was made showing there may be superior sagittal sinus thrombosis, which was diagnosed by digital subtraction angiography (DSA). The patient bled and had superior sagittal sinus thrombosis. The incidence of superior sagittal sinus thrombosis among ITP patients is even rare. This article clarifies the clinical experiences of ITP complicated with paradoxical disease thrombosis. We summarize its relevant mechanisms, suggesting that ITP patients have the risk of severe bleeding due to thrombocytopenia, but paradoxically, they also have the risk of thrombotic disease, so as to remind clinicians to pay attention to the potential thromboembolic risk and avoid thromboembolism in ITP treatment.

## 2. Case report

**Figure 1.**
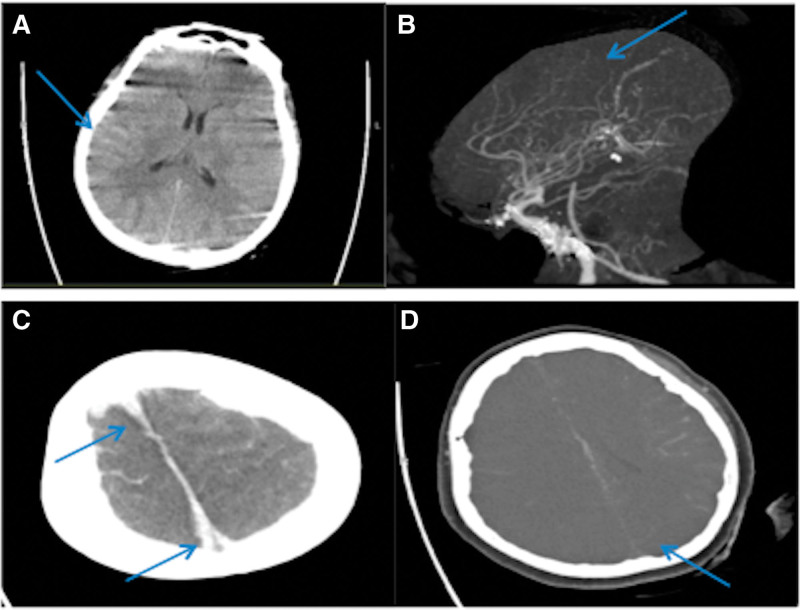
(A) Brain CT showed subarachnoid hemorrhage. (B) Brain CTA showed that no obvious dilatation, stenosis, or tumor-like lesions were found. In the cerebral artery circle, anterior cerebral arteries, middle cerebral arteries, and posterior cerebral arteries on both sides and their branches. Superior sagittal sinus and its surrounding veins were poorly displayed, so superior sagittal sinus thrombosis was considered. (C) Brain CTV showed the density of the superior sagittal sinus increased and brain edema occurred on CT scan. Superior sagittal sinus thrombosis was considered. (D) Brain CTV showed poor development of intracranial veins. CT = computed tomography, CTA = computed tomographic angiography, CTV = computed tomographic venography.

A 32-year-old female patient was found with thrombocytopenia in November 2020 with a platelet count of 12 × 10^9^/L. Her bone marrow examination showed megakaryocytes increased and the function of platelet production was poor, so she was diagnosed with ITP; 20 mg prednisone was given, with the platelet count increasing to 40 × 10^9^/L. In January 2021 her platelet count was checked again at 20 × 10^9^/L. The patient changed to take 24 mg methylprednisolone and cyclosporine for treatment. She stopped to take the medicines by herself with a platelet count of 80 × 10^9^/L in March 2021.On April 22, 2021, the patient was treated with immunoglobulin for 5 days and 80 mg methylprednisolone because of ecchymosis in both lower limbs and gingival bleeding with a platelet count of 1 × 10^9^/L. Then methylprednisolone was reduced to 16 mg once a day later, and the platelet count was maintained at 60 × 10^9^/L. On August 15, 2021, the patient had diffuse petechiae and ecchymosis of limbs and gums continuously bleeding. And then vaginal bleeding began with a large number of blood clots, and she had black stool 2 to 3 times 1 day. She felt dizzy. So the patient was admitted to the Hematology Department of our hospital on August 23, 2021. At that time her red blood cell count was 2.44 × 10^12^/L, hemoglobin was 60 g/L, and platelet count was 1 × 10^9^/L. Therefore she was given 80 mg once a day methylprednisolone, 0.4 g/kg for 5 days of immunoglobulin, and 25 mg of Eltrombopag, a thrombopoietin receptor agonist (TPO-RA), once a day to help platelet creating. She also took hemostatic drugs, red blood cells, and platelet suspension. But her vagina was still bleeding, so she was given 5 g norethisterone tablets (estrogen) once every 8 hours and 150 mg cyclosporine twice 1 day orally on September 6, 2021. On September 10, 2021, the patient had persistent headaches and vomiting. Brain CT showed subarachnoid hemorrhage (Fig. 1A). She was given hemostasis treatment, but the headache continued to be aggravated. At 00:15, on September 15, 2021, the patient suddenly vomited with a level 3 headache and obvious temporal pain on both sides. The muscle strength of the left limb was in grade 1, and the left palm was in a “claw” posture. The left superficial sensation weakened, the left Babinski sign was positive, and her right limb was shaking irregularly. The subarachnoid hemorrhage was basically in the same condition as the previous CT result. Neurosurgeon considered that the patient had vasospasm after subarachnoid hemorrhage, and likely to have cerebral infarction which might be venous sinus thrombosis. According to the patient’s clinical manifestations, it is considered that the patient had increased intracranial pressure, so she received intracranial pressure reduction treatments, such as mannitol dehydration, maintenance of systolic pressure >110 mm Hg, bed head elevation of 30°. Seizures can aggravate intracranial pressure, so we gave antiepileptic treatment to the patient.^[2]^ Ten minutes later, the patient suddenly lost consciousness and convulsed, with a closed jaw and breath shortage. Therefore she was given endotracheal intubation and at 4:31 she was transferred to the intensive care unit. Her Glasgow coma scale (GCS) was 1 + T + 1, her pupils were normal and sensitive to light reflection. There was no resistance in her soft neck, and the rest of the nervous system examinations were uncoordinated. The antiepilepsy, inhibited vasospasm, and reduced intracranial pressure were continuously performed. At 05:28 the patient had dilated left and right pupils successively and the bilateral light reflection disappeared. The brain CTA showed that there was no obvious dilatation, stenosis, or tumor-like lesions in the cerebral artery circle, and in both sides of anterior cerebral arteries, middle cerebral arteries, posterior cerebral arteries, and basilar arteries and their main branches. But the display of the superior sagittal sinus was poor, so there may be thrombosis (Fig. 1B). Then further head CTV was made showing in Scan way that the density of the superior sagittal sinus increased. And the intracranial vein of CTV was poorly developed. The cerebral falx, bilateral tentorium cerebellum, lateral fissure cistern, and frontal and parietal lobe were mainly filled with linear high-density shadow. Most of the cerebral sulcus disappeared, and the ventricular system was narrow. There were multiple high-density shadows in the sulci of both cerebral hemispheres which may be cortical venous thrombosis. She also had brain edema. The image suggested the possibility of thrombosis (Fig. 1C and D). Further examinations were made with platelet counts at 33 × 10^9^/L and D-dimer at 20 ug/mL. Her blood gas showed metabolic acidosis and hypernatremia. She had DSA and venous sinus thrombectomy. DSA showed the internal carotid arteries on both sides were tortuous, the forward blood flow of the right internal carotid artery was slow and ended at the beginning of the M1 segment of the ipsilateral middle cerebral artery and the beginning of the A1 segment of the anterior cerebral artery; the anterior blood flow of the left internal carotid artery was slow and ended at the M1 segment of the ipsilateral middle cerebral artery and the A1 segment of the anterior cerebral artery, and there was no deep perforators development; the anterior blood flow of the left vertebral artery was slow and ended at the end of V3 segment. The above image showed an extremely increasing intracranial pressure, but scalp vein dilatation and obvious staining of dura in both cerebral hemispheres were not founded, suggesting obstruction of superior sagittal sinus reflux. Because it was difficult to successfully remove the thrombus, The patient‘s family members refused to take any treatments and the patient was discharged.

## 3. Discussion

This patient was complicated with thrombotic diseases on the basis of immune thrombocytopenic diseases, resulting in fatal consequences. We reviewed the literatures on mechanisms of 2 antinomy diseases for physicians to make better diagnosis treatments.

ITP is characterized by the autoantibody-mediated destruction of platelets and the impaired production of platelets from megakaryocytes,^[3]^ resulting in the decrease of platelet count (<100 × 10^9^/L),^[4]^ with clinical manifestations of various bleeding tendencies, which is more likely to occur when platelets count <30 × 10^9^/L.^[1]^ However, it has been reported that patients with this hemorrhagic disease have a risk of thrombosis and embolism in recent years. The annual incidence of venous thrombosis is (6.5–6.9)/1000, and that of arterial thrombosis is (14.7–15.0)/1000.^[5]^ Thrombosis may occur even when the platelet count is low.^[6]^

The risk mechanism of thrombosis in ITP involves many factors such as ITP itself,^[7]^ its treatments,^[2]^ and the patient’s constitution, medical history, and former medication.^[8]^

ITP has potential thrombosis and embolism risk. It can give rise to thrombophilic factors,^[9]^ such as immune disorders and the increase of immature platelets. It may be partly related to platelets' pathological complement activation, resulting in vascular inflammation and thrombosis.^[10]^ Moreover, the increase of red cell microparticles and platelet microparticles with high thrombogenic potential and the increased of endothelial cell autoantibody damage may also play a role in thrombosis.^[11,12]^ In addition, the lifespan of ITP platelets is significantly shortened, and the proportion of large platelets and young platelets among patients with chronic ITP is higher, in which there may be more thrombotic activities.^[13]^

ITP-related treatments include glucocorticoids, TPO-RA, intravenous immunoglobulins, immunosuppressant applications, and splenectomy also contributes to thrombosis.^[14–17]^ Drugs may cause arterial vasospasm, increase plasma viscosity and promote thrombosis by inducing platelets and endothelial cell activation, increas ing the level of coagulation factors and reducing the level of antithrombotic factors (Table 1 presents the thrombogenic mechanism of drugs combined with this patient’s medication).^[18–28]^

**Table 1:** Thrombogenic mechanism of ITP treatment drugs combined with this patient’s medication.

Drugs	Mechanism
Glucocorticoid Cyclosporine (calcineurin inhibitor)	Glucocorticoids can increase the level of plasma thromboxane and reduce the level of antithrombotic factors, and they can inhibit fibrinolytic activity.^[18–20]^ Cyclosporine can change the structure of platelets membrane and the conformation of glycoprotein IIb IIIA; it can also change the intracellular calcium flow in lymphocytes and increase the cytoplasmic calcium concentration in platelets, resulting in the expression of fibrinogen receptor and platelet activation. Lymphocytes and platelets share surface antigens and play a role in platelet aggregation.^[21,22]^
Immunoglobulin	Immunoglobulin causes arterial vasospasm and increases blood viscosity, and some immunoglobulin preparations contain activated coagulation factor XI.^[23,24]^
TPO-RA	TPO-RA induces the proliferation and differentiation of megakaryocyte precursor cells in bone marrow, increasing the peripheral platelet count.^[25–27]^
Estrogen	Estrogen causes the increase of coagulation factors level, the decrease of coagulation inhibitor level, and the inhibition of fibrinolysis.^[28]^

ITP = immune thrombocytopenia, TPO-RA = thrombopoietin receptor agonists.

Splenectomy may also increase the risk of thrombosis. When splenectomy is done, damaged cells and microsomes with procoagulant effect constantly persist in the blood, resulting in the transformation of vascular homeostasis to enhanced coagulation. It is of high possibility to cause thrombosis with the exposure of plasma cholesterol (aggravating arteriosclerosis) and C-reactive protein.^[29]^

The patients who have thrombogenesis risk factors are those at high age, have a previous history of thrombosis, long-term bed rest, and owning complications (such as heart diseases, obesity, diabetes, etc), and take oral estrogen or contraceptives, etc.^[30]^

In this case, the patient was treated with glucocorticoid and cyclosporine. During this period, the patient stopped taking medicine by herself. Later, she was bleeding. The platelet count was found to be 1 × 10^9^/L. She was treated with immunoglobulin, TPO-RA, cyclosporine, and a large number of glucocorticoids. Because of vaginal bleeding, estrogen and hemostatic drugs were added. All the above medications could cause thrombosis. Although her platelet count was lower than the normal value and she was bleeding, there were still thrombotic events. There are hidden dangers of thrombosis among ITP patients relating to the ITP itself and its therapeutic drugs. Clinicians should be vigilant against thrombosis while bleeding tendency occurs during the treatment. When making the treatment plan, we should fully consider whether the plan is necessary and understand the advantages and disadvantages of each treatment plan. For patients with thromboembolism risks, we should inform them of the risks and make thrombosis screening and prevention early.

It is hard to determine the management of thrombotic events, and the initiation and duration of anticoagulant therapy among ITP patients. Anticoagulant therapy will be individualized to be short-term or long-term depending on the type of thrombus and the existence of reversible thrombotic risk factors. Thrombus recurrence should be prevented too. In the course of anticoagulant, the platelet count should be maintained at a threshold of at least 50 × 10^9^/L. For ITP patients with thrombotic events and bleeding symptoms or a platelet count of <30 × 10^9^/L, they should be given low-dose or full-dose anticoagulant therapy according to the severity of thrombosis, the response history of ITP therapy and the severity of bleeding.^[31]^

In recent years, it has been reported that deep venous thrombosis (53.7%) and pulmonary embolism (11.82%) are common in ITP patients. Other parts’ morbidity lists as follows such as arterial thrombosis (2.11%), mesenteric venous thrombosis (2.31%), portal vein thrombosis (2.27%), splenic vein thrombosis (0.3%), hepatic vein thrombosis (0.18%), while cerebral venous sinus thrombosis is only 0.47%.^[32]^

Cerebral venous sinus and cerebral venous thrombosis (CVT) is a group of cerebrovascular diseases, collectively referred to as CVT.^[33]^ Among them, the incidence of cerebral venous sinus thrombosis is low, accounting for about 0.5 to 1% of all cerebrovascular diseases.^[34]^ Superior sagittal sinus thrombosis is the most common site of noninfectious venous sinus thrombosis.^[35–37]^

It is difficult to recognize CVT in ITP patients. The incidence of CVT among ITP patients is even rarer, and there are only a few cases reported in domestic and foreign literature.

The main risk factors of CVT are as follows:

1. Blood hypercoagulable state.
2. Hereditary abnormal coagulation mechanism.
3. Hemodynamic abnormalities (thrombocytopathy, primary polycythemia, iron deficiency anemia, disseminated intravascular coagulation).
4. Drug-induced (oral contraceptives, cortisol hormone, and androgen).
5. Infection or tumor infiltration (temporal phlebitis, cancerous dura mater).^[38–43]^

CVT causes obstruction of cerebral blood reflux or disturbance of cerebrospinal fluid circulation. Its clinical manifestations are intracranial hypertension and/or focal brain damage. Headache is one of the common manifestations which can be serious and persistent, accompanied by projectile vomiting. Patients can also have papilledema and are prone to epilepsy. Epilepsy is the second clinical symptom in addition to headache.^[39,44–46]^ The patient had risks of thrombosis during the course of onset and treatments and showed the characteristics of CVT after onset. The former mentioned blood diseases and immune system diseases can induce CVT. This patient first presented with the symptoms of headache, which was more severe and gradually aggravated, and the patient had nausea and vomiting, which was in the form of jet, and subsequently, the patient had decreased consciousness level, dilated pupil and disappeared light reflex, and irregular breathing which were indications of high criminal pressure.^[47]^ The suspected slight subarachnoid hemorrhage was shown in CT. However, the headache was not relieved and aggravated continuously. Later, the muscle strength decreased significantly, and muscular tension increased. She also had convulsions, closed teeth, and other epileptic symptoms. Her GCS was low. Brain CTA ruled out the problem of intracranial arteries and it showed a poor display of superior sagittal sinus, suggesting thrombosis occurred. The CTV examination was made. The scan showed that the density of the superior sagittal sinus was increased. And the intracranial vein of CTV was poorly developed, suggesting the possibility of thrombosis, which was finally diagnosed by DSA. For ITP patients with intracranial hypertension symptoms such as headache, vomiting, or epilepsy, we should pay high attention to the occurrence of CVT. The imaging examination methods of cerebral venous system imaging include DSA, CTV, and magnetic resonance venography (MRV), providing important imaging findings for intracranial vein and intracranial venous sinus lesions. DSA is the gold standard for diagnosis. In CT, some patients show high-density triangles or high-density cords. Secondly, it can also have many indirect signs of CVT, such as shallower cerebral sulcus, diffuse brain tissue swelling, white matter low density, and brain parenchyma damage such as venous infarction, hemorrhage, or hemorrhagic cerebral infarction. The imaging findings of CTV and MRV mainly showed interruption of venous continuity and filling defect area which are the direct signs and the most direct evidence for the diagnosis of CVT. In addition, CTV and MRV can also show thickening of the distal venous sinus and dilation of the drainage vein. Indirect signs such as infarction, hemorrhage, or swelling of brain tissue can be seen in the corresponding brain tissue. DSA showed poor or no development of the corresponding vein.^[48,49]^ And sometimes CTA will also be important in the diagnosis of CVT.

Timely treatment is related to the outcome of the disease. The treatment of controlling intracranial pressure is very important in the prevention of cerebral hernia. The general method is hypertonic therapy (mannitol or hypertonic saline dehydration treatment).^[50]^ The head of the bed should be raised to 30°, so that the vena jugularis intera will not be compressed, and the drainage of cerebral veins is also very convenient.^[51]^ Temperature rise affects intracranial pressure by increasing brain metabolic demand and cerebral blood flow, so body temperature should be controlled below 37°C.^[52]^ Because high blood pressure maintains cerebral perfusion, it is necessary to maintain systolic blood pressure >110 mm Hg.^[53]^ Epilepsy can increase the metabolic rate of the brain to aggravate intracranial hypertension, so anti-epileptic treatment is very necessary.^[54]^ Respiratory dysfunction is common in patients with intracranial hypertension, and PaCO2 can cause cerebral artery contraction by alkalizing cerebrospinal fluid. Therefore, patients with GCS <8 or respiratory failure should receive early mechanical ventilation and maintain an appropriate level of positive end-expiratory pressure.^[55]^ Non-synchronous mechanical ventilation and excitement of patients will increase intrathoracic pressure, resulting in reduced thoracic venous reflux, thus cerebral blood volume and intracranial pressure increase. Therefore, appropriate analgesia and sedation should be given.^[56]^ In the process of adjuvant treatment, the treatment of primary disease is also extremely important. Anticoagulation therapy, such as heparin or warfarin, thrombolysis therapy such as urokinase or alteplase, and interventional thrombolysis are the treatment measures.^[40,57–59]^ Treatments, in this case, were to reduce intracranial pressure. When she lost consciousness, she was given mechanical ventilation and systolic blood pressure was maintained, which improved cerebral blood supply, and anti-epilepsy, and made interventional thrombolysis. It was difficult to successfully remove the thrombus, and the patient failed to be treated successfully. Therefore, it is more important to identify and diagnose CVT in ITP patients in the early period. In the process of diagnosis, intracranial pressure should be controlled in time, and organ support treatment should be carried out to prepare for the subsequent thrombolysis and thrombus removal, and the thrombus should be treated early to save lives.

## 4. Conclusion

ITP is not only a hemorrhagic disease but also a thrombotic disease. Therefore thrombus monitoring and screening should be carried out, and early prevention or appropriate anticoagulant treatment should be selected, especially for those patients with high risk factors. Low platelet count can not prevent thrombus. This case is an ITP treatment manifestation suggesting that the clinicians should not only pay attention to the bleeding tendency but thrombosis formation and its risks, so as to provide personalized and best treatments for patients. When the patient has recurrent headaches accompanied by intracranial hypertension symptoms with no obvious abnormality or only bleeding on CT, it is necessary to consider the possibility of CVT. The clinicians should timely complete craniocerebral CTA, CTV , MRV, or further conduct DSA to make a clear diagnosis. After diagnosis, timely treatment should be carried out soon to prevent further aggravation of neurological damage symptoms.

## Author contributions

**Supervision:** Ge Zhang, Jinggang Ding.

**Writing – original draft:** Yuhui Wang.

**Writing – review & editing:** Yuhui Wang.
